# Expression Analysis of Ermin and Listerin E3 Ubiquitin Protein Ligase 1 Genes in Autistic Patients

**DOI:** 10.3389/fnmol.2021.701977

**Published:** 2021-07-19

**Authors:** Shadi Shiva, Jalal Gharesouran, Hani Sabaie, Mohammad Reza Asadi, Shahram Arsang-Jang, Mohammad Taheri, Maryam Rezazadeh

**Affiliations:** ^1^Pediatric Health Research Center, Tabriz University of Medical Sciences, Tabriz, Iran; ^2^Department of Medical Genetics, Faculty of Medicine, Tabriz University of Medical Sciences, Tabriz, Iran; ^3^Student Research Committee, Tabriz University of Medical Sciences, Tabriz, Iran; ^4^Cancer Gene Therapy Research Center, Zanjan University of Medical Science, Zanjan, Iran; ^5^Skull Base Research Center, Loghman Hakim Hospital, Shahid Beheshti University of Medical Sciences, Tehran, Iran

**Keywords:** autism spectrum disorders, *ERMN*, *LTN1*, expression, biomarker

## Abstract

Autism spectrum disorder (ASD) is a severe neurodevelopmental disorder that involves social interaction defects, impairment of non-verbal and verbal interactions, and limited interests along with stereotypic activities. Its incidence has been increasing rapidly in recent decades. Despite numerous attempts to understand the pathophysiology of ASD, its exact etiology is still unclear. Recent data shows the role of accurate myelination and translational regulation in ASD’s pathogenesis. In this study, we assessed Ermin (*ERMN*) and Listerin E3 Ubiquitin Protein Ligase 1 (*LTN1*) genes expression in Iranian ASD patients and age- and gender-matched healthy subjects’ peripheral blood using quantitative real-time PCR to recognize any probable dysregulation in the expression of these genes and propose this disorder’s mechanisms. Analysis of the expression demonstrated a significant *ERMN* downregulation in total ASD patients compared to the healthy individuals (posterior beta = −0.794, adjusted *P*-value = 0.025). *LTN1* expression was suggestively higher in ASD patients in comparison with the corresponding control individuals. Considering the gender of study participants, the analysis showed that the mentioned genes’ different expression levels were significant only in male subjects. Besides, a significant correlation was found between expression of the mentioned genes (*r* = −0.49, *P* < 0.0001). The present study provides further supports for the contribution of *ERMN* and *LTN1* in ASD’s pathogenesis.

## Introduction

Autism spectrum disorder (ASD) is a disorder in neurodevelopment that occurs early in life (Parellada et al., 2014). ASD includes poor interpersonal communication, social interaction, and repetitive behaviors (Carlisi et al., 2017). Although an incidence of 1 per 160 kids has been reported throughout the world (Elsabbagh et al., 2012), some varieties are observed in the epidemiology of disorder in distinct world areas (Elsabbagh et al., 2012; Baxter et al., 2015). These variations could be attributed to different techniques, variations in diagnostic or community identification, and probable risk factors (Rice et al., 2012). According to the Diagnostic and Statistical Manual of Mental Disorders (DSM-5), ASD includes two classes of symptoms, the first of which is poor communication and social interaction, and the second is repetitive and restricted behavior (American Psychiatric Association, 2013). Therefore, ASD is currently defined as autistic disorder, Asperger’s disorder, pervasive developmental disorder not otherwise specified (PDD-NOS), Rett’s disorder, and childhood disintegrative disorder. This broad-spectrum causes diversity in terms of the diagnosis of ASD regarding the previous version of DSM (Harstad et al., 2015). Although the pathophysiology of ASD is not clear, it is assumed that developmental abnormalities in the limbic region, frontal lobe, and putamen lead to imbalanced inhibiting and exciting of neurons. Recently, researchers have revealed that disrupted neuronal connectivity could be associated with alterations in the neurons related to the production of white matter and myelination in different regions of the brain of ASD patients (Galvez-Contreras et al., 2020). Moreover, the disruption of brain translational regulation in ASD progression is increasingly being taken into consideration. New mechanisms, such as ribosome stalling and ribosome quality control (RQC), are highly related to translational regulation. Consequently, all these might play a role in the pathogenesis of these disorders (Hui et al., 2019). According to the literature about blood transcriptome in patients with ASD, extraction of valuable information about the pathophysiology of the disease is possible using peripheral blood. In other words, the best way of assessing systemic alterations is blood investigation because of the role in controlling both centrally and peripherally (Ansel et al., 2017). The etiology of ASD is complicated, heterogeneous, and multifactorial. Diverse environmental factors related to the intrauterine environment and genetic-related risk factors, along with abnormalities in molecular and cellular signaling pathways, might contribute to the etiology of the disorder (Parellada et al., 2014). There has been extended research on autism genetics in recent decades, and findings are increasingly being reported (Thapar and Rutter, 2020).

An oligodendrocyte-specific protein that plays a role in myelination is encoded by Ermin (*ERMN*) or Juxtanodin. This protein is expressed in a late phase of myelination and is located in the myelin sheath’s outer cytoplasmic lip and the paranodal loops in mature neurons (Brockschnieder et al., 2006). Correct myelination is vital in ASD (Zikopoulos and Barbas, 2010; Broek et al., 2014). Listerin E3 Ubiquitin Protein Ligase 1 (*LTN1*) encoded protein is a part of the RQC complex. The latter complex plays a role in the degradation of the polybasic-mediated stalled protein (Bengtson and Joazeiro, 2010; Brandman et al., 2012; Defenouillère et al., 2013; Verma et al., 2013). These two genes were selected to further investigate their possible role and interaction in ASD pathogenesis.

Since ASD is a prevalent disorder with a rapidly elevating incidence in previous decades and due to the possible role of *ERMN* and *LTN1* genes in the pathogenesis of this disorder through contributing to disturbances related to oligodendrocytes and RQC pathway disruption and the possible usage of these components in diagnosis, prognosis, and early treatment, we investigated the expression of *ERMN* and *LTN1* performed by qRT-PCR in the peripheral blood of ASD patients.

## Materials and Methods

### Participants and Samples

We carried out this case-control study on 100 participants, including 50 ASD patients and 50 healthy controls matched for age and gender. The current study was approved by the committee of clinical research ethics of Tabriz University of Medical Sciences (Ethical code: IR.TBZMED.REC.1399.1017). The case group participants were recruited from the Tabriz Children’s Hospital, Iran. A specialized psychiatrist confirmed the diagnosis of all patients based on the DSM-5 (American Psychiatric Association, 2013). The exclusion criteria entailed being affected by other comorbid genetic syndromes or metabolic disorders. All individuals or their surrogates signed written informed consent, and peripheral blood samples of 5 ml were taken from the participants.

### Expression Assays

We used a published methodology from our group (Ghafouri-Fard et al., 2021). Total RNA was extracted from whole blood using Hybrid-R^TM^ Blood RNA purification kit (GeneALL, Seoul, South Korea) according to the manufacturer’s protocol. Assessment of the extracted RNA in terms of quantity and quality was performed by NanoDrop (Thermo Scientific, Wilmington, DE, United States). The synthesis of cDNA was done by the cDNA synthesis Kit (GeneALL) according to the manufacturer’s instructions. The obtained cDNA was stored at −20°C for further investigation. The designing of specific probes and primers for *ERMN*, *LTN1*, and *HPRT1* was carried out applying Allele ID 7 software (Premier Biosoft, Palo Alto, CA, United States). *HPRT1* was chosen because it has been introduced as one of the most stable housekeeping genes in autism studies (Anitha et al., 2012; Nardone et al., 2014; Zhou et al., 2016). PCR program comprised an initial activation phase for 5 min at 94°C, and 40 cycles at 94°C for 10 s and 60°C for 40 s. Total reaction volumes were 20 μL containing 4 μl of cDNA, 3.5 μl double distilled water, 10 μl of Master Mix 2×, 250 and 900 nM concentrations of probe and each primer, respectively. Table 1 summarizes probes and primers sequences. The qPCR was carried out by the Step OnePlus^TM^ Real-Time PCR and the RealQ Plus2x Master Mix (Ampliqon, Odense, Denmark).

**TABLE 1:** Primers and probes used for expression assays.

**Gene name**	**Gene reference ID**	**Primer sequences (5′-3′)**	**Probe sequences**
*ERMN*	NM_001304344.2 NM_001304345.2 NM_001009959.3 NM_001304346.2 NM_020711.3	Forward primer TGTTGCCTTTATGCTTTCAAACTG Reverse primer TCTGCTGCCCACCAATCTTC	AGCCCCTCCCAGTGTCAACCTCAC
*LTN1*	NM_001320766.2 NM_015565.3 XM_006723987.4 XM_017028316.2	Forward primer CGCTCAGCTTATTTTGAGTTAGTC Reverse primer GGGTCACTGTCATCAATGCTAAG	CTGCATTGTGCCAGCGCATTCCAC
*HPRT1*	NM_000194.3	Forward primer AGCCTAAGATGAGAGTTC Reverse primer CACAGAACTAGAACATTGATA	CATCTGGAGTCCTATTGACATCGC

### Statistical Analysis

Data were analyzed using Stan, “ggplot2,” “brms,” and pROC packages in the R v.4 software. *ERMN* and *LTN1* genes’ relative expressions were compared in the patients with ASD and the healthy subjects besides their subgroups employing the Bayesian regression model. The effects of gender and age were adjusted. Adjusted *P*-values were reported. Expression of mentioned genes was also compared between different age groups as well as between males and females. Correlations between the study variables were appraised using Pearson correlation coefficients. The diagnostic power of genes was measured using receiver operating characteristic (ROC) curve analysis.

## Results

### General Demographic Data

Demographic and clinical data of patients and healthy subjects are summarized in Table 2.

**TABLE 2:** Demographic data of ASD children and controls.

**Variables**	**ASD cases**	**Controls**
Male/Female [no. (%)]	32 (64%)/18 (36%)	31 (62%)/19 (38%)
Age (year and mean ± SD)	6.4 ± 1	6.3 ± 0.7
Age range (year)	3.5–9	3.5–8
Age at onset (mean ± SD, year)	3.2 ± 0.8	–

### Expression Assays

**FIGURE 1:**
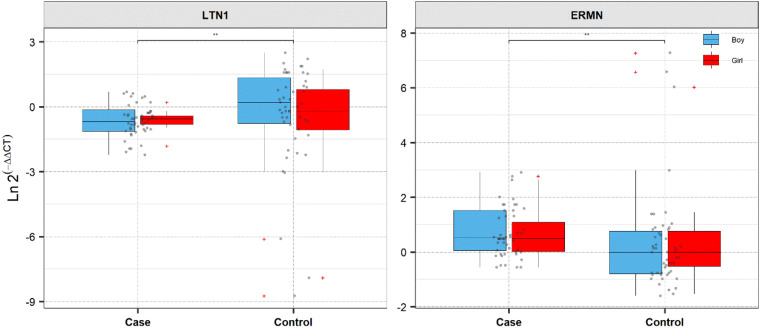
Expression of *ERMN* and *LTN1* in cases and controls’ blood samples. Ln (Efficiency^Δ*Ct*^) method was used to calculate expression levels of genes, and the values are depicted as black dots. Means of expression levels and interquartile range are displayed.

Figure 1 shows *ERMN* and *LTN1* genes’ relative expression levels in patients with ASD and controls.

The *ERMN* gene’s expression was suggestively decreased in total ASD cases compared with total controls (posterior beta = −0.794, adjusted *P-*value = 0.025). Such decreased expression was found between boy subgroups too (posterior beta = −0.938, adjusted *P-*value = 0.041). On the contrary, *LTN1* was higher in total ASD cases vs. controls (posterior beta = 1.317, adjusted *P-*value = 0.033). Such increased expression was identified in boy cases compared to boy controls (posterior beta = 1.269, adjusted *P-*value = 0.01). Age of total and boy individuals were associated with *LTN1* gene’s expression levels. Tables 3, 4 show the detailed data about the relative expression of *ERMN* and *LTN1*, respectively.

**TABLE 3:** Relative levels of *ERMN* in ASD cases and controls according to the Bayesian quantile regression model.

	***ERMN***	**Posterior beta of 2^(–*ddct)*^**	**SE**	Adjusted *P*-value*	**95% Crl for beta**
**Total**	Group, case vs. control	−0.794	0.16	0.025	(−1.12, −0.5)
	Sex, girl vs. boy	0.039	0.18	0.78	(−0.31, 0.37)
	Age (years)	−0.031	0.1	0.981	(−0.23, 0.14)
	Group × gender	0.336	−0.22	0.876	(−0.22, 1.08)
Boy	Case vs. control	−0.938	0.2	0.041	(−1.34, −0.54)
	Age	−0.028	0.12	0.729	(−0.25, 0.2)
Girl	Case vs. control	−0.509	0.3	0.172	(−1.1, 0.08)
	Age	−0.078	0.16	0.369	(−0.43, 0.23)

*Estimated from frequentist methods.

*ASD, autism spectrum disorder; CrI, credible interval; SE, standard error.*

**TABLE 4:** Relative levels of *LTN1* in ASD cases and controls according to the Bayesian quantile regression model.

	***LTN1***	**Posterior beta of 2^(–*ddct)*^**	**SE**	Adjusted *P*-value*	**95% Crl for beta**
Total	Group, case vs. control	1.317	0.26	0.033	(0.78, 1.82)
	Sex, girl vs. boy	−0.238	0.24	0.393	(−0.69, 0.25)
	Age (years)	−0.244	0.08	0.048	(−0.4, −0.09)
	Group × gender	−0.13	0.42	0.409	−0.98, 0.67)
Boy	Case vs. control	1.269	0.29	0.01	(0.69, 1.84)
	Age	−0.278	0.09	0.045	(−0.48, −0.1)
Girl	Case vs. control	1.08	0.51	0.328	(0.16, 1.98)
	Age	−0.15	0.14	0.64	(−0.45, 0.11)

*Estimated from frequentist methods.

*ASD, autism spectrum disorder; CrI, credible interval; SE, standard error.*

### Correlation Analysis

**FIGURE 2:**
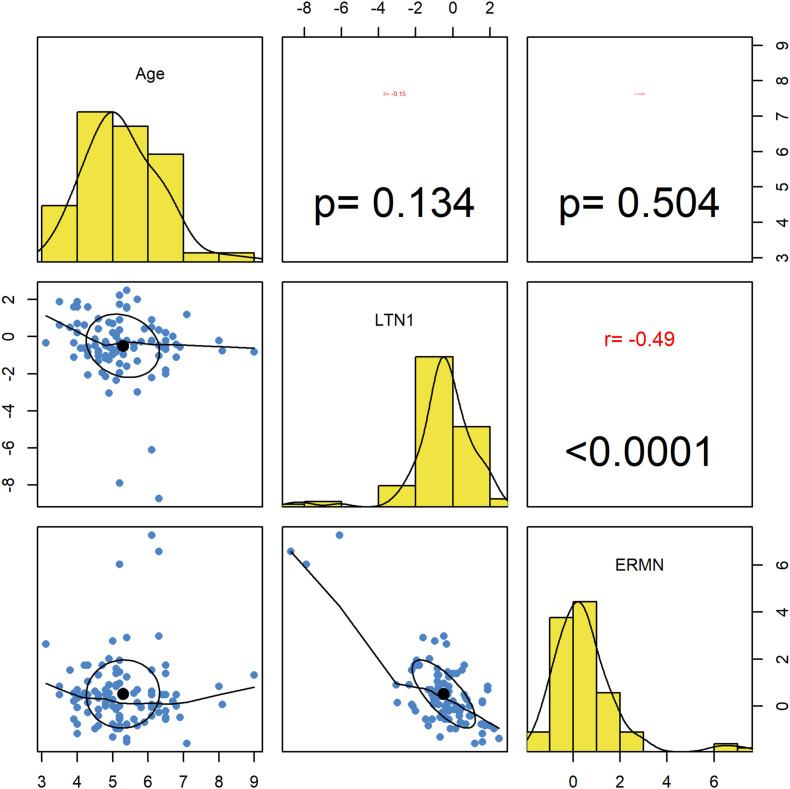
Correlations between *ERMN* and *LTN1* genes’ expression levels and age. The distribution of variables is presented on the diagonal. The lower portion of the diagonal shows bivariate scatter plots with a fitted line. The upper part of the diagonal demonstrates correlation coefficients and *P*-values.

Ermin and *LTN1* expression was not correlated with participants’ age. Significant opposite correlations were detected between assessed genes’ expression levels (*r* = −0.49, *P* < 0.0001) (Figure 2).

### ROC Curve Analysis

**FIGURE 3:**
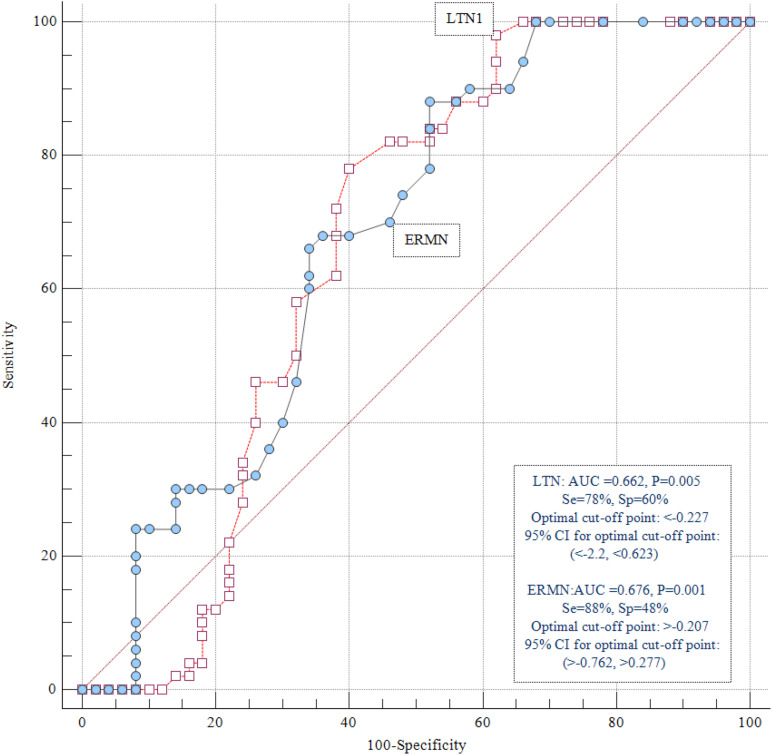
ROC curve analysis. *ERMN* and *LTN1* transcript levels displayed diagnostic power of 0.676 and 0.662, respectively.

We assessed the diagnostic power of *ERMN* and *LTN1* to distinguish ASD patients from healthy controls at different threshold settings. *ERMN* and *LTN1* transcript levels presented diagnostic power of 0.676 and 0.662, respectively, based on the area under the ROC curves (Figure 3).

## Discussion

Autism spectrum disorder is among the disorders with the highest heritability with the typically multifactorial origin. Strong interest exists in using gene discoveries to achieve insights into ASD’s underlying biology (Quesnel-Vallieres et al., 2019). In the present study, we evaluated *ERMN* and *LTN1* expression in the peripheral blood of ASD patients and healthy individuals.

Lower levels of *ERMN* were detected in patients with ASD in comparison with the controls. *ERMN* encoded protein belonging to the ezrin-radixin-moesin family (ERM) was identified as a cytoskeletal protein in 2006. It is demonstrated that this gene stimulating differentiation of oligodendroglial cells and the maintenance of myelin sheath *via* interaction with myosin phosphatase Rho interacts with protein (Mprip/p116RIP) and inactivates RhoA, which is a GTPase regulating the cytoskeleton rearrangement in differentiating cells (Wang et al., 2020). Remarkably, *ERMN* is located inside the *AUTS5* locus boundaries, which has been repeatedly shown to be linked with autism (Maestrini et al., 2010), language impairment (Bartlett et al., 2004), and IQ (Posthuma et al., 2005). A preceding study showed the overexpression of *ERMN* in a patient presented with rare genetic variants (meSNV). Furthermore, targeted resequencing showed that higher loads of *ERMN* were recognized in ASD patients with rare damaging mutations. Function gaining *via* overexpression and function losing *via* deleterious mutations suggests gentle gene dosage equilibrium (Homs et al., 2016). Besides, deletions including *ERMN* were reported in impaired communication and developmental delay patients (Newbury et al., 2009). Expression of *ERMN* is lower in patients with epilepsy which suggests its role in the epileptogenic process (Wang et al., 2011). Moreover, investigations showed the upregulation of *ERMN* in the prefrontal cortex (Martins-de-Souza et al., 2009a) and its downregulation in the anterior temporal lobe of SCZ cases’ samples. The downregulation of *ERMN* may explain why the integrity of the white matter is disrupted in individuals with SCZ (Martins-de-Souza et al., 2009b). Besides, a study suggested that reduced *ERMN* expression in the leukocytes could cause demyelination in patients with relapsing-remitting MS (Salek Esfahani et al., 2019).

Conversely, the level of *LTN1* was higher in patients with ASD in comparison with controls. *LTN1* gene acts in a specific pathway for protein quality control by mediating the incomplete polypeptides’ proteolytic targeting produced by ribosome stalling (Martin et al., 2020). The potential mechanism of the *LTN1* gene in the pathogenesis of ASD is possibly *via* disruption of the pathway of RQC, which is shown to influence brain function and is involved in ASD pathogenic processes (Hui et al., 2019). To the best of our knowledge, the present study is the first report on the expression of *LTN1* in ASD patients’ peripheral blood. Only one preceding study recommended according to (1) the essential genes’ importance in the etiology of ASD as presented by their role in the critical stages of development and the increased burden of rare, damaging mutations in patients with ASD; (2) their co-expression with the ASD’s high-confidence genes in the brain; and (3) the genetic evidence suggesting from the transmission and *de novo* association (TADA) analysis that *LTN1* gene is among the strongest candidates whose roles should be evaluated in ASD (Ji et al., 2016).

Furthermore, gender-related differences were found in *ERMN* and *LTN1* genes’ expression levels (Tables 2, 3). The absence of difference in these genes’ expression in female patients might be caused by gender-based differences found in the phenotypes or underlying mechanism of ASD. According to previous reports, affected boys and girls showed phenotypic differences (Van Wijngaarden-Cremers et al., 2014). For example, repetitive and stereotyped behaviors were found to be less common in girls compared with boys (Bölte et al., 2011). These differences are attributable to the underlying mechanisms of sexual dimorphism, such as those associated with the dosage of sex chromosomal genes and the levels of sex hormones (Van Wijngaarden-Cremers et al., 2014).

The present study results suggest that the expression of *LTN1* is associated but not correlated with the age of total participants and male participants (Table 3 and Figure 2). Additional studies are required to be further investigate this relationship and interpret such results. Besides, a significant inverse correlation was found between *ERMN* and *LTN1* levels, which shows an interactive network, probably owing to the translational regulation. Moreover, the sex-related differences in *ERMN* and *LTN1* expression (Tables 2, 3) suggest the effect of sex-related factors on this pathway. A robust male bias has been disclosed in ASD with striking consistency, though no mechanism is found yet to account for these sex differences conclusively (Werling and Geschwind, 2013).

Also, we evaluated the *LTN1* and *ERMN* genes’ diagnostic power to differentiate ASD patients from healthy individuals and stated the diagnostic power of 0.676 and 0.662 for *ERMN* and *LTN1*, respectively. These data should be interpreted with caution due to the relatively small sample size of the present study. If future research supports the present study’s findings, the transcription level of *ERMN*/*LTN1* could be regarded as an ASD disease marker in male patients. Moreover, replicating these experiments in cerebrospinal fluid samples would provide some evidence that the differences in whole blood transcript levels are markers for changes in transcript levels in the nervous system. Taken together, the present study further verified *LTN1* and *ERMN* genes’ putative role in ASD’s pathophysiology, which potentiates the genes as functional studies’ candidates.

## Data Availability Statement

The raw data supporting the conclusions of this article will be made available by the authors, without undue reservation.

## Ethics Statement

The studies involving human participants were reviewed and approved by Tabriz University of Medical Sciences (IR.TBZMED.REC.1399.1017). Written informed consent to participate in this study was provided by the participants’ legal guardian/next of kin.

## Author Contributions

MT and MR wrote the draft and revised it. SA-J analyzed the data. JG, HS, SS, and MA performed the experiment and data collection. All authors contributed to the article and approved the submitted version.

## Conflict of Interest

The authors declare that the research was conducted in the absence of any commercial or financial relationships that could be construed as a potential conflict of interest.
